# Limitations of and Solutions to Using 6 mm Corneal Spherical Aberration and Q Value after Laser Refractive Surgery

**DOI:** 10.3390/bioengineering11020190

**Published:** 2024-02-16

**Authors:** Sung Ho Choi, Yeo Kyoung Won, Sung Jin Na, DeokJo Nam, Dong Hui Lim

**Affiliations:** 1 First Samsung Eye Clinic, Seoul 06621, Republic of Korea; ophth-choi@daum.net (S.H.C.); said8@naver.com (S.J.N.); londjve@naver.co (D.N.); 2 Department of Ophthalmology, Samsung Medical Center School of Medicine, Sungkyunkwan University, Seoul 06351, Republic of Korea; wyk900105@hanmail.net; 3 Samsung Advanced Institute for Health Sciences and Technology, Sungkyunkwan University, Seoul 06351, Republic of Korea

**Keywords:** multifocal IOL, corneal spherical aberration, LASIK, PRK, Q value, corneal asphericity

## Abstract

This study aimed to evaluate the spherical aberration (SA) in different corneal areas before and after femtosecond laser-assisted in situ keratomileusis (fLASIK) and transepithelial photorefractive keratectomy (tPRK), with the goal of identifying the limitations of and potential improvements in using SA within a 6 mm area. The study included 62 patients who underwent fLASIK and tPRK. Complete eye examinations including keratometry, corneal epithelial thickness, central corneal thickness, and topography were performed preoperatively and postoperatively. Anterior, posterior, and total corneal aberrations were measured preoperatively and three months postoperatively, with pupil diameters ranging from 2 to 8 mm. In the fLASIK group, compared to the preoperative SA, the anterior and total SA increased postoperatively in the 6 and 7 mm areas. In the tPRK group, meanwhile, the anterior and total SA of the 5 mm or larger areas increased postoperatively. An area of 6 mm or larger showed an increase in correlation with the changes in Q value and refractive correction. As the corneal SA and asphericity in the 6 mm zone cannot specifically demonstrate the status of areas smaller than 6 mm or changes in the optical zone after laser refractive surgery, comparison with normal values in various areas of the cornea is necessary.

## 1. Introduction

The use of excimer laser refractive surgery for treating myopia has become popular over the past 20 years [1]. As an increasing number of patients who underwent laser-assisted in situ keratomileusis (LASIK) and photorefractive keratectomy (PRK) 20–30 years ago age, the incidence of cataract development in these individuals is now on the rise [2,3]. Cataracts, a natural part of the aging process that leads to a decrease in vision, require surgery to restore vision. Many of these patients who have previously undergone LASIK or PRK are eager to maintain their glasses-free lifestyle after cataract surgery and therefore they often request the implantation of multifocal intraocular lenses (IOLs) rather than conventional monofocal IOLs [4,5,6].

Corneal high-order aberrations (HOAs) play a crucial role in the success and quality of vision after multifocal IOL implantation [7,8,9]. Spherical aberrations (SA) are one of the most significant HOAs that reduce retinal image quality and also affect the subjective refraction of defocus [10]. As the glare, halos, and starburst phenomena observed by patients with multifocal IOLs can be attributed to spherical aberration [11,12], it is essential to evaluate HOAs including SA before deciding whether to implant a multifocal IOL. 

Correcting myopia increases the root mean square of HOAs, especially the SA of the cornea [13]; values obtained through a 6 mm diameter are frequently used as the testing area [5,9]. Since the normal cornea exhibits positive SA, most multifocal IOLs are designed with negative SA to counteract the positive SA found in the central 6 mm of the normal cornea. However, in cases where refractive surgery has been performed, SA may increase more than in a normal cornea [13]. Possible reasons for increased SAs after refractive surgery are reported by several studies [10]. Holladay et al. [14] demonstrated that LASIK surgery for myopia correction leads to an increase in corneal asphericity, resulting in a vision decrease. Similarly, both Jimenez et al. [15] and Gatinel et al. [16,17] also represented the change in corneal asphericity following myopic laser refractive surgery. Anera et al. [18] found that higher levels of myopia are associated with a greater increase in corneal asphericity and a subsequent decrease in visual outcomes. Changes in corneal asphericity are a key factor in explaining some of the induced SAs, given that corneal asphericity significantly influences the amount of aberration when the corneal curvature radius is same [10]. This means that, even after IOL surgery, not all of the SA is offset and a positive SA remains in a post-refractive eye. Therefore, eyes that have undergone greater myopic correction will retain more positive SA after multifocal IOL implantation, potentially leading to poorer visual outcomes. However, most studies have reported good outcomes with multifocal IOLs [19,20].

Using a 6 mm diameter to measure SA may be insufficient for accurately assessing the corneal state after refractive surgery, as the surgeries can alter the corneal shape in ways that extend above the central 6 mm zone. Moreover, observing corneal topography maps to examine SA changes post refractive surgery is not always straightforward, given the complexity of interpreting the maps. Therefore, to address these issues, it is necessary to evaluate corneal SA in different areas and establish reference values for these areas when selecting multifocal IOLs.

This retrospective, non-randomized study aimed to evaluate changes in SA in different corneal areas before and after femtosecond laser-assisted in situ keratomileusis (fLASIK) and transepithelial photorefractive keratectomy (tPRK) for myopia correction, with the goal of identifying the limitations and potential improvements of using SA within a 6 mm area.

## 2. Methods

### 2.1. Patients and Study Design

**Figure 1:**
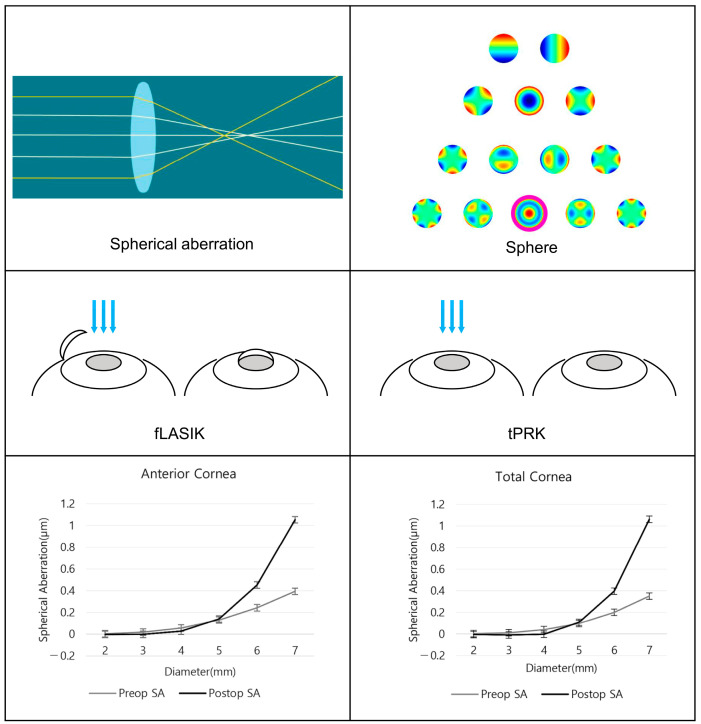
A schematic picture of the study.

This retrospective, non-randomized study enrolled consecutive patients who underwent fLASIK and tPRK for myopia correction, including astigmatism, at the First Samsung Eye Clinic. All patients included in the study underwent laser refractive surgery at least three months prior to January 2022. The surgical procedure was decided after consultation with the patient, considering the patient’s eye condition. All patients were provided with detailed information about the study and the risks and benefits of the surgery, and signed informed consent forms to participate. The study was designed to evaluate changes in SA in different corneal areas before and after fLASIK and tPRK (Figure 1). This study was approved by the ethics committee of the Institutional Review Board of the First Samsung Eye Clinic (No. FSEC-202304-HR-007-01), and adhered to the guidelines of the Declaration of Helsinki.

### 2.2. Inclusion and Exclusion Criteria

Patients older than 18 years with stable refraction for at least 12 months were included. The preoperative spherical equivalent of the manifest refraction was less than 0 diopters. In addition, soft contact lens wear was discontinued for at least one week, and rigid contact lens wear was discontinued for at least one month prior to the preoperative examination. The exclusion criteria were abnormal or keratoconic topography, previous ocular surgery, concurrent ocular diseases, and systemic diseases that could affect corneal wound healing.

### 2.3. Ocular Examinations

Complete eye examinations, including uncorrected (UDVA) and corrected (CDVA) distance visual acuity, slit-lamp examination of the anterior segment of the eye, fundus examination using wide-field digital imaging (Optomap, Optos Inc., Marlborough, MA, USA), intraocular pressure, keratometry, and Shiempflug-based corneal tomography (Pentacam; Oculus, Wetzlar, Germany), were performed preoperatively. Pentacam measurements were performed three times or more, confirming a mean K difference within 0.25 diopters between the test results. The results from the Zernike map of the first examination were summarized. Corneal epithelial thickness was measured using anterior segment optical coherence tomography (Cirrus HD-OCT, Zeiss, Dublin, CA, USA), and central corneal thickness (CCT) was measured using an ultrasonic pachymeter (US-500, NIDEK, Tokyo, Japan). Postoperative examinations three months after surgery included UDVA and CDVA, slit-lamp examination of the cornea, and corneal topography, with the same procedures as used preoperatively. Anterior, posterior, and total corneal spherical aberrations were measured preoperatively and three months postoperatively, with pupil diameters ranging from 2 to 8 mm. using a Pentacam. The preoperative and postoperative examination results, conducted before and three months after the surgery, were compared.

### 2.4. Surgical Procedure

Topical anesthesia (0.5% proparacaine hydrochloride (Alcaine)) eye drops, Alcon, Fort Worth, TX, USA) were administered before the surgical procedure. For fLASIK, a corneal flap with a diameter of 8.8 mm was cut at a depth of 100 µm using a femtosecond laser (Femto LDV Z8; Ziemer, CH-2562 Port, Switzerland), and the stroma was ablated using an EX500 excimer laser (Wavelight, Alcon, Fort Worth, TX, USA) guided by the Custom-Q algorithm. The flap was carefully repositioned after the laser treatment. A bandage contact lens (Acuvue^®^ 1-Day, Johnson & Johnson, Jacksonville, FL, USA) was applied to avoid flap displacement and was removed the next day. For tPRK, one-step ablation was performed using the transepithelial PRK mode (StreamLight^®^, Wavelight, Alcon, Fort Worth, TX, USA). Mitomycin C 0.02% was applied for 40 s, followed by copious irrigation with cold balanced salt solution (BSS). Bandage contact lens (Acuvue^®^ 1-Week, Johnson & Johnson, Jacksonville, FL, USA) was applied during the epithelial healing period and removed after 4–5 days. The optical diameter was set at 6.5 mm in all eyes, and the residual corneal stroma thickness was no less than 300 µm. The postoperative medication regimen for patients who underwent fLASIk and tPRK included the use of 0.02% fluorometholone eye drops four times daily for two weeks and three months, respectively. In addition, all patients were instructed to use 0.5% moxifloxacin eye drops four times daily for two weeks and 0.15% sodium hyaluronate eye drops every two hours for three months.

### 2.5. Statistical Analysis

Data were analyzed using SPSS (version 28.0; SPSS, Inc., Chicago, CA, USA). The normality of data was tested using the Kolmogorov–Smirnov test. As the data were normally distributed, *t*-tests were used to compare pre-and postoperative values according to the size of the inspection diameter, and a *p*-value of 0.05 or less was considered statistically significant.

## 3. Results

This study included 124 eyes from 62 patients who underwent fLASIK and tPRK for correction of myopia, including astigmatism. In total, 46 eyes underwent fLASIK, and 78 eyes underwent tPRK. Table 1 presents the baseline patient characteristics and Figure S1 shows the pre- and postoperative axial map of a fLASIK patient. 

Table S1 shows the outcomes of the pre- and postoperative refractive outcomes, SimK, and 6 mm Q values. When comparing the refractive error, SimK, and Q values before and after surgery, there were statistically significant differences (all *p*-values < 0.001). However, when the refractive error, SimK, and Q values were compared between the fLASIK and tPRK groups, no statistically significant differences were observed except for the postoperative Q value (*p*-value = 0.023).

**Figure 2:**
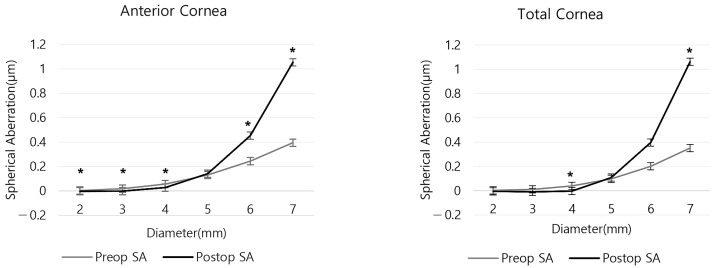
Anterior and total corneal spherical aberration by corneal region before and after refractive surgery. (* *p* < 0.05 between preoperative and postoperative SA).

**Figure 3:**
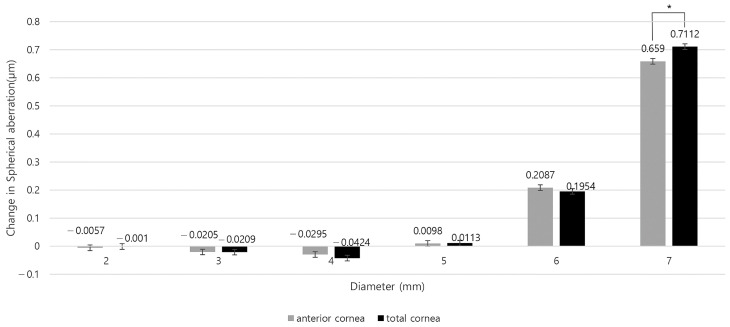
Changes in anterior and total corneal spherical aberration by corneal region before and after refractive surgery. (* *p* < 0.05 between preoperative and postoperative SA).

As shown in Table S2, the preoperative anterior and total SA were positive in all areas. However, anterior SA before and after surgery showed statistically significant differences in all areas except for the 5 mm zone (all *p* < 0.05, except for *p* = 0.232 in the 5 mm zone). For the total SA before and after surgery, there were statistically significant differences in the 4 and 7 mm zones (*p* = 0.023 and <0.001, respectively). The mean anterior SA in the 2 and 3 mm zones and the mean total SA in the 3 and 4 mm zones were negative postoperatively (Figure 2 and Table S2). The two SAs increased postoperatively in the 5, 6 and 7 mm zones, and decreased in areas less than 4 mm (Figure 3 and Table S2). 

**Figure 4:**
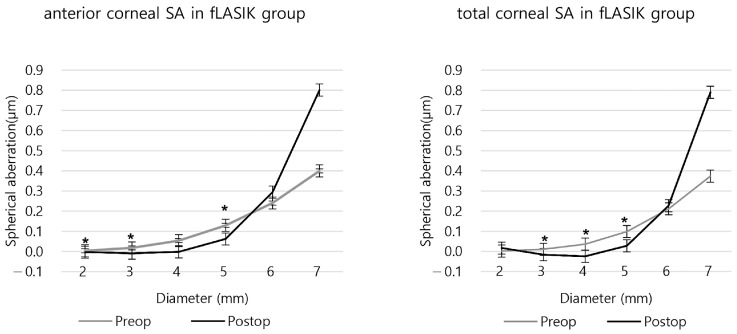
The anterior and total corneal spherical aberration before and after fLASIK. (* *p* < 0.05 between preoperative and postoperative SA).

In the fLASIK group, significant changes in the anterior SA were observed before and after surgery in the 5 mm or smaller areas, except for the 4 mm area (*p* = 0.228). For the total SA, except for the 2 mm zone (*p* = 0.535), there were significant differences. Postoperative anteriors were negative in the 2–4 mm area and total SAs were negative in the 3 and 4 mm areas, respectively (Figure 4 and Table S2). Compared to the preoperative SA, the anterior and the total SA increased after surgery in the 6 mm, 7 mm areas, and the 2 mm area of the total SA also increased postoperatively (Figure 4 and Figure S2). 

**Figure 5:**
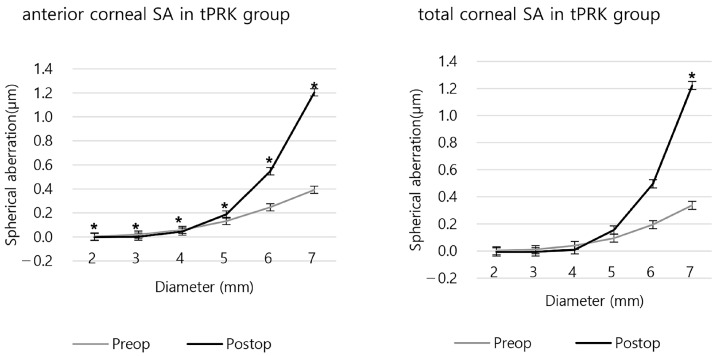
The anterior and total corneal spherical aberration before and after tPRK. (* *p* < 0.05 between preoperative and postoperative SA).

In the tPRK group, postoperative SA was negative in 2 mm of the anterior SA and 2 and 3 mm of the total SA. (Figure 5 and Table S2). Compared to the preoperative SA, the anterior and the total SA of the 5 mm or larger areas increased after surgery, while the 4 mm or smaller SA decreased (Figure 5 and Figure S2).

When comparing the two groups, significant differences in the changes in corneal SA before and after surgery were found in all areas, except for 2 mm for the anterior SA and 4 mm or smaller areas for the total SA (Table S2).

### Relationship between the Change in Spherical Aberration and the Amount of Myopic Correction and Change in Q Value

The amount of myopic correction and the change in the Q value of 6 mm showed a proportional relationship after the surgery (R^2^ = 0.6985) (Figure S3). The correlation between the surgically induced changes in the Q value and area-specific SA showed a similar trend to that between the changes in refractive correction and SA. As shown in Figure S4, both the anterior and the total corneal SA in across areas measuring 5 mm or smaller showed a relatively small change after surgery, regardless of myopic correction, while an area of 6 mm or larger showed an increase in correlation with the changes in Q value and refractive correction. 

## 4. Discussion

Corneal HOAs, particularly SAs, significantly impact vision quality and success following multifocal IOL implantation. SA not only reduces retinal image quality but also influences subjective refraction. Given that patients who underwent laser refractive surgery have more positive SAs after multifocal IOL implantation compared to those with no prior surgery, accurate assessment of SA is critical before proceeding with multifocal IOL implantation. Since conventional 6 mm diameter measurements for SA may not fully show corneal changes post-refractive surgery, we aimed to thoroughly evaluate the corneal SA across different areas pre- and post-refractive surgery. This study showed that it is crucial to compare patient SAs against the normal values in various areas of the cornea, instead of depending solely on measurements within the 6 mm zone.

The degree of myopia correction was positively associated with an increase in the 6 mm SA and Q values after laser refractive surgery. However, despite the increase in 6 mm SA, multifocal IOLs can provide good visual acuity in many patients, indicating the limitations of using SA as a measure of corneal status in the 6 mm region. When surgery was performed with a 6.5 mm optical zone, the SA increased proportionally to the degree of myopic correction in the test area above 6 mm. This proportional increase suggests a predictable pattern of corneal change according to a degree of correction. Conversely, there was no difference or decrease in the preoperative level below 5 mm. In daily life, because pupils are typically approximately 3 mm in diameter, the sudden increase in SA that occurs beyond 6 mm does not significantly affect visual quality except in very dark conditions. As we age, pupil size tends to decrease [21]; therefore, the relative value of the 6 mm SA in eyes that have undergone refractive surgery is reduced when selecting an IOL. If the optical zone narrows after refractive surgery and the SA changes abruptly within a 4 mm diameter, visual acuity may also be impaired in normal pupils. 

Therefore, to select an appropriate multifocal IOL, the corneal SA corresponding to the patient’s maximum and mesopic pupils should be measured. The value of the mesopic pupil can be used to evaluate the quality of vision in everyday life, and that of the maximum pupil size can predict visual impairment during nighttime driving and in low-light conditions, which can be used for IOL selection and preoperative consultation. However, only the 6 mm SA of the IOL optic is publicly available. It would be better if the SA for each area of the IOL could be disclosed so that it could be compared with the corneal SA of the patient’s daily pupil size; however, this is not the case. Instead, a comparison could be made between the SA of a normal cornea and that of a patient’s cornea. Using this method, a small amount of normal corneal data was used to investigate the SA of corneas that had undergone LASIK surgery. The results showed that there was little to no difference in the SA between the central region of the LASIK-treated corneas and that of a normal cornea. The sample size was increased in order to obtain statistically significant data. Currently, it is possible to compare a patient’s SA data with those of a normal cornea using a graph of the SA of the normal cornea, as shown in Figure S5a. If a patient’s SA in the central 2–5 mm region is similar to the normal graph, then it may be acceptable to choose an IOL with a negative SA.

To utilize the results of this study, we collected and compared cases of low vision caused by irregular corneal astigmatism after multifocal IOL implantation. Correcting the corneal SA through topography-guided LASIK improved visual acuity [9]. Another case involved a patient who had undergone radial keratotomy (RK) and received a diffractive IOL in one eye and an extended depth-of-focus IOL in the other eye. However, visual acuity did not improve in either eye, and topography-guided LASIK was performed, resulting in a decrease in the 3–5 mm SA and the restoration of 20/20 visual acuity in both eyes (Figure S5). It has been reported that the results of multifocal IOL implantation are not favorable in eyes with previous RK [22], as Jesper et al. reported that the 4–6 mm RMSh is twice as high as that in PRK, especially with a high Sab [22]. This case demonstrates that increased corneal SA after multifocal IOL implantation may be the cause of low vision, and that correcting it with topography-guided LASIK can improve visual outcomes.

The Q value, which represents the shape of the cornea, is closely related to the corneal SA because it determines the optical characteristics of the cornea. As shown in Figure S2, there was a positive correlation between the 6 mm Q value and the amount of myopic correction. Typically, Q values greater than 0 indicate an oblate shape, 0 indicates a spherical shape, and −0.52 to 0 indicates a prolate shape. Generally, when myopia is corrected using LASIK or PRK, the cornea becomes more oblate, leading to an increase in SA. However, as shown in Figure S4c, the 6 mm Q value does not accurately reflect the SA in the central optical zone of the cornea, and the regression graph showing changes in SA indicates that an optical zone of 5 mm or less is likely to maintain a prolate shape. Although the Pentacam provides Q values greater than 6 mm, Q values of less than 5 mm cannot be obtained. However, similar to the SA, Q values below 5 mm were expected to be similar to those in normal eyes. Therefore, caution is necessary when using the 6 mm Q value after refractive surgery, similar to the 6 mm SA.

Although the optical zone was set to 6.5 mm for all eyes, the peripheral corneal SA, including the 6 mm zone, increased. This phenomenon can be explained by the cosine effect, which occurs because the ablation depth per pulse decreases as it moves away from the center of the excimer laser beam. Yoon et al. [10], using a computational model, explained that, if the ablation depth per pulse is constant, myopic correction can result in a negative SA. This may be a contributing factor to the decreased 4 mm SA after surgery in this study. It was found that using an SA measurement for a zone at least 1.5 mm narrower than the applied 6.5 mm optical zone reduced the impact of the cosine effect. Additionally, tPRK showed a more abrupt change in smaller areas than fLASIK, indicating the need to consider SA in various areas depending on the surgical method and optical zone size. Previous studies have reported side effects such as increased night glare, regression, and decentration when the optical zone is narrowed [23,24,25,26]. Therefore, caution should be exercised in patients with large pupils by considering topography-guided LASIK or a wider optical zone to reduce SA.

The magnitude of the SA can differ depending on the type of measuring device used, even for the same eye. Specifically, the SA value measured by a slit-scanning or a Placido disc-based topography are generally found to be lower than that measured by Scheimpflug imaging devices such as Galilei^TM^ (Zeimer, Port, Switzerland) or Pentacam. As the SA measured by the dual Scheimpflug imaging Galilei is higher than that measured by the single Scheimpflug tomography of Pentacam, it is advisable to determine the normal range of each device before use [27,28]. For instance, the normative range of SA for Galilei is between +0.15 µm and +0.30 µm [29]. In this study, the total corneal SA for 6 mm was 0.201 ± 0.083 µm (mean ± SD), and the range of two standard deviations was 0.035 µm to 0.367 µm.

To accurately represent the postoperative state of the cornea after refractive surgery, it is essential to present the SAs for each region, from the central 2 mm to 6–7 mm. The commonly used 6 mm region is useful for identifying the cause of visual impairment when the pupil is larger than 6 mm after laser refractive surgery or in normal corneas. However, in eyes that have undergone laser refractive surgery, the 6 mm SA and Q values may vary depending on the amount of defocus correction, making it difficult to interpret the data intuitively. When comparing the results of various refractive surgeries, the values in the 6 mm region lose their value as data because of the bias of the defocus correction and the ablation pattern. Comparing a patient’s data with the normal range, which is indicated by values such as two standard deviations or a 95% confidence interval, is essential for an accurate assessment.

One limitation of our study is that we did not obtain a range of abnormal corneal SA that was unsuitable for multifocal IOL implantation. Analyzing the interaction between corneal SA and various types of multifocal IOLs requires consideration of multifocal IOL-cornea interactions, the number of corneal SA, and pupil size changes under different lighting, which necessitates the use of simulation programs and optical bench tests. Moreover, the samples in this study were obtained from young individuals who had not undergone cataract surgery, and the data were analyzed within one year of undergoing refractive surgery. We did not analyze the corneas of individuals who developed cataracts because we believe that performing refractive surgery on corneas with normal HOAs would better reflect changes in the SA than on corneas with high HOAs due to degenerative changes. We believe that data from young corneas have clinical value because, when comparing the SA of patients with low vision after inserting a multifocal IOL with the data from this study, the difference was often resolved through topography-guided LASIK or PRK [9].

Corneal topography provides information about the shape of the cornea, which is crucial to understanding its function. However, each topographic map has unique characteristics and may not accurately represent the optical features of the cornea. To supplement this, asphericity and HOA data are frequently used. The results of this study indicate that the corneal SA and asphericity in the 6 mm zone can indicate the extent to which a cornea deviates from the standard cornea but cannot specifically demonstrate the status of areas smaller than 6 mm or changes in the optical zone after surgery. Unfortunately, users may fail to utilize these indices properly because of the habitual use of limited testing areas. The continued use of a 6 mm area value as a standard metric in many papers might be attributed to the limited data provided by the measuring equipment and a lack of knowledge and understanding among ophthalmologists regarding their interpretation and utilization. Finally, this study highlights the need for a method to compare the obtained corneal SA and asphericity in various areas of the cornea with normal values and to confirm changes before and after surgery to address the limitations of existing methods [30,31,32].

## 5. Conclusions

This is the first study to evaluate changes in SA in different corneal areas before and after fLASIK and tPRK, with the goal of identifying the limitations of and potential improvements in using SA within a 6 mm area. An area of 6 mm or larger showed an increase in correlation with the changes in Q value and refractive correction. The corneal SA and asphericity in the 6 mm zone cannot specifically demonstrate the status of areas smaller than 6 mm or changes in optical zone after laser refractive surgery. Accurately assessing the postoperative state of the cornea following refractive surgery necessitates a comprehensive analysis of SAs across different areas, rather than relying solely on measurements within the 6 mm zone. It is crucial to compare patient data against the normal values in various areas of the cornea.

## Figures and Tables

**Table 1:** Baseline characteristics of patients who underwent fLASIK and tPRK.

Patients, N (Eyes)	62 (124 Eyes)
M:F	24:38
Age (min,max) (year)	27.88 (18~43)
Follow-up periods (min,max) (month)	5.65 ± 2.34 (3~11)
fLASIk:tPRK (eyes)	46:78

fLASIK: femtosecond laser-assisted in situ keratomileusis; tPRK: transepithelial photorefractive keratectomy.

## Data Availability

The data that support the findings of this study are available on request from the corresponding author. The data are not publicly available due to privacy or ethical restrictions.

## Supplementary Materials

The following supporting information can be downloaded at: https://www.mdpi.com/article/10.3390/bioengineering11020190/s1, This article includes the Supplementary Figures S1–S5 and the Supplementary Tables S1 and S2; Figure S1: An example of the axial map before and after fLASIK; Figure S2: Changes in spherical aberration by region before and after fLASIK and tPRK; Figure S3: Correlation between the amount of myopia correction and the change in Q value (6 mm) caused by the surgery; Figure S4: Correlation between change in corneal spherical aberration and myopia correction amount or change in Q value (6 mm); Figure S5: A case of a patient with previous radial keratotomy (RK) who underwent topography-guided LASIK because of poor visual acuity after implantation of multifocal IOLs; Table S1: The outcomes of the preoperative and postoperative refractive outcomes, SimK, and 6 mm Q values; Table S2: Results of anterior corneal spherical aberration and total corneal spherical aberration before and after surgery in each area.
