# Supplementary material for: Limitations of and Solutions to Using 6 mm Corneal Spherical Aberration and Q Value after Laser Refractive Surgery
Source: Bioengineering (Basel). 2024 Feb 16;11(2):190. doi: 10.3390/bioengineering11020190 (PMC10886312; doi:10.3390/bioengineering11020190)
Supplement: Supplementary file 1 [file bioengineering-11-00190-s001.zip › bioengineering-2827792-supplementary.pdf]

Figure S1. An example of the axial map before and after fLASIK. a) the axial map of pentacam before fLASIK. b) the axial map of pentacam at 3 months after fLASIK.

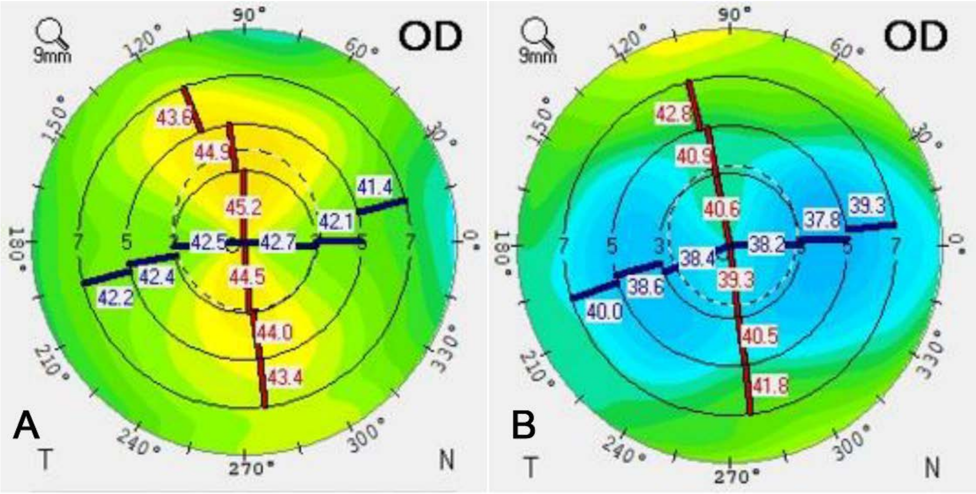

Figure S2. Changes in spherical aberration by region before and after fLASIK and tPRK

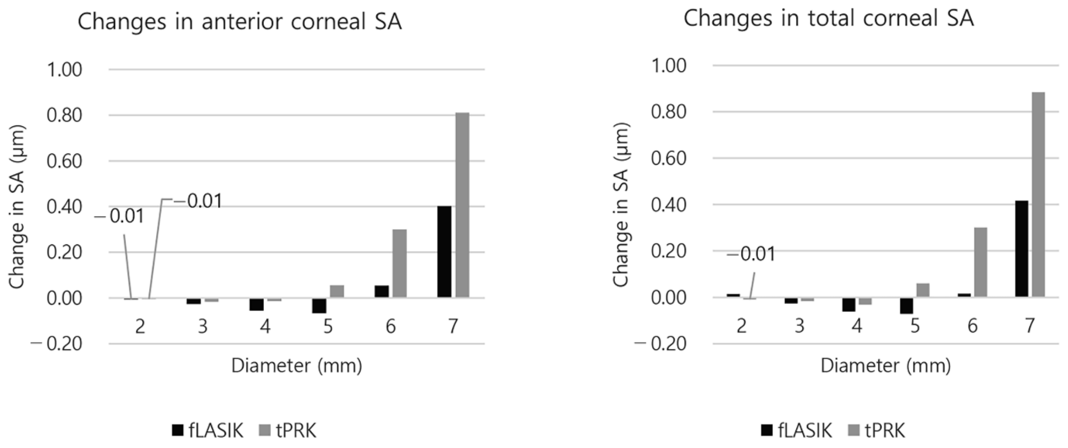

Figure S3. Correlation between the amount of myopia correction and the change in Q value (6 mm) caused by the surgery.

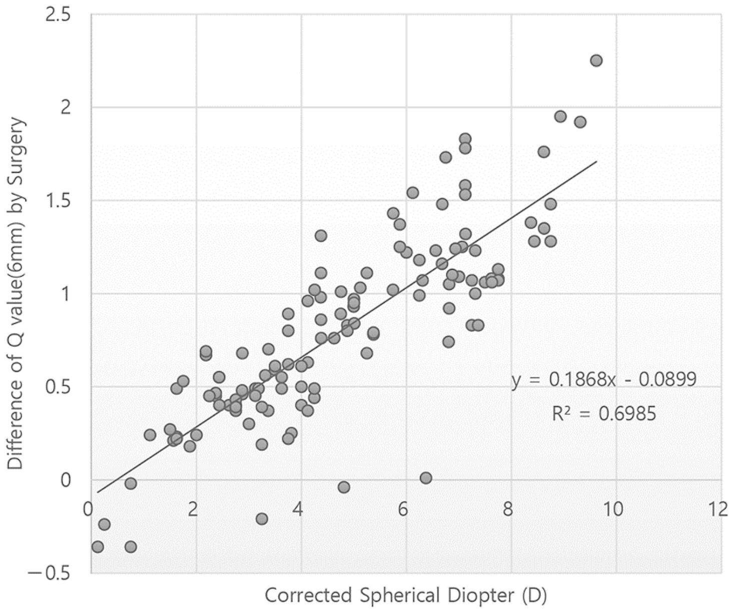

Figure S4. Correlation between change in corneal spherical aberration and myopia correction amount or change in Q value(6mm) .a) correlation between myopia correction amount and change in anterior corneal spherical aberration. b) correlation between myopia correction amount and change in total corneal spherical aberration. c) correlation between change in Q value(6 mm) and change in total corneal spherical aberration

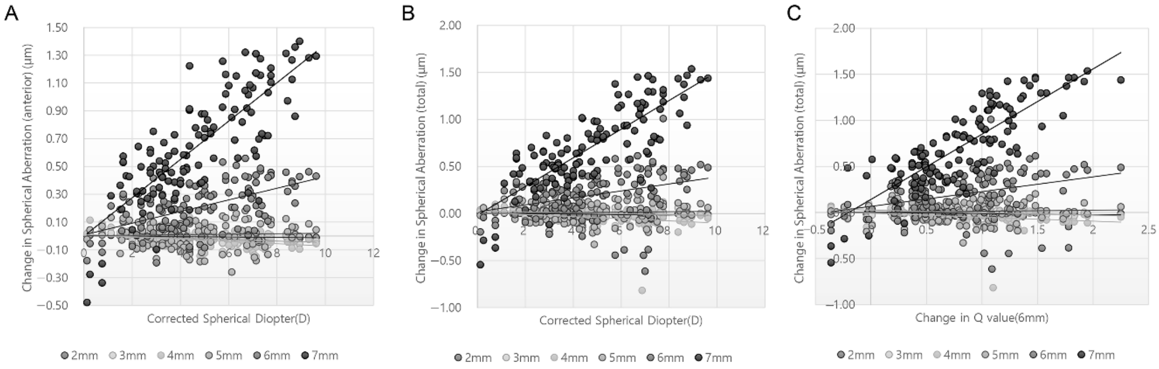

Figure S5. A case of a patient with previous radial keratotomy (RK) who underwent topography-guided LASIK because of poor visual acuity after implantation of multifocal IOLs

a) The red and blue solid lines on the graph represent the spherical aberration (SA) of the anterior cornea in a patient who underwent radial keratotomy. Compared to the average in a normal eye (purple line), a significant difference was observed from 4 mm onwards. Despite receiving multifocal IOL implantation in both eyes, distance visual acuity did not improve beyond 0.8 in the right eye and 0.7 in the left eye, and J5 for near vision. Forty days after receiving femtosecond laser-assisted topography-guided LASIK (topoLASIK), the 2-5 mm SA improved (dotted line), the uncorrected visual acuity in both eyes improved to 20/20, and J2 for near vision. b shows the appearance at the time of the first visit, and c and d show the axial maps before and after topoLASIK.

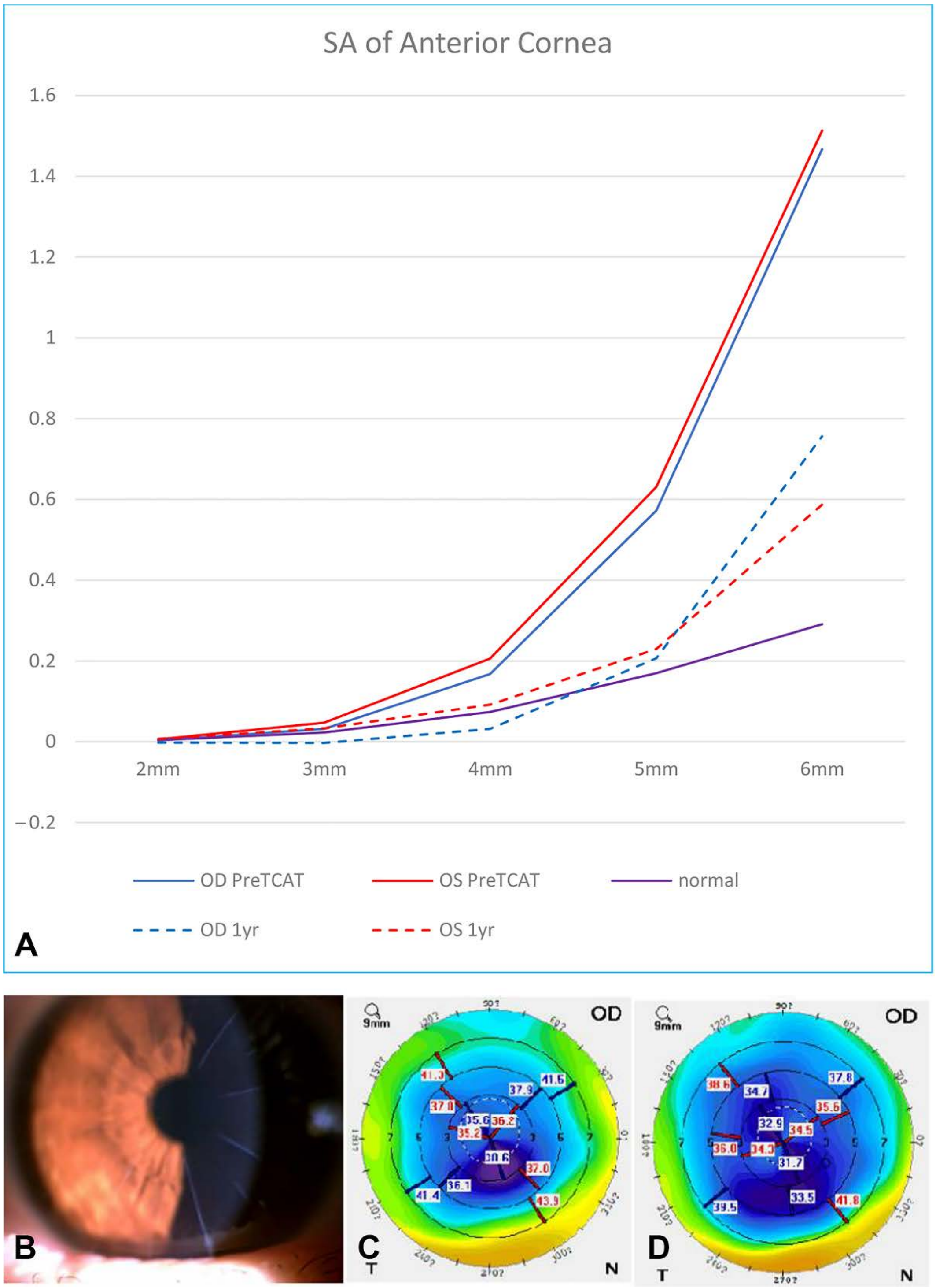
SA of Anterior Cornea
1.6
1.4
1.2
1
0.8
0.6
0.4
0.2
0
−0.2
2mm
3mm
4mm
5mm
6mm
OD PreTCAT
OS PreTCAT
normal
OD 1yr
OS 1yr
A
B
C
D
OD
OD
9mm
9mm
T
N
T
N

Table S1. The Outcomes of the Preoperative and Postoperative refractive outcomes, SimK, and 6mm Q Values

	**Preoperative**	**Postoperative**	***P value****
Spherical equivalent(D)	-4.78 ± 2.22 (-9.62~-0.13)	-0.24 ± 0.53 (-1.38 ~ 1.25)	**.000**
Spherical diopter(D)	-4.09 ± 2.29 (-8.87 ~ 2.00)	-0.040 ± 0.57 (-1.25 ~1.75)	**.000**
Cylindrical diopter(D)	-1.38 ± 1.01 (-4.25 ~ 0)	-0.42 ± 0.35 (-1.5 ~ 0)	**.000**
Flat K(D)	42.27 ± 3.69 (39.2 ~ 46)	39.00 ± 2.18 (33.6 ~ 43.3)	**.000**
Steep K(D)	44.46 ± 1.92 (36.3 ~ 49.5)	39.84 ± 2.31 (34.6 ~ 43.9)	**.000**
Q value (6 mm)	-0.34 ± 0.16 (-0.67 ~ -0.11)	0.46 ± 0.51 (-0.68 ~ 1.9)	**.000**
fLASIK			
Spherical equivalent(D)	-4.06 ± 2.28 (-9.62 ~ -0.13)	-0.45 ± 0.47 (-1.38 ~ 0.38)	**.000**
Spherical diopter(D)	-3.36 ± 2.52 (-8.87 ~ 2.00)	-0.26 ± 0.47 (-1.25 ~ 0.75)	**.000**
Cylindrical diopter(D)	-1.40 ± 1.05 (-4.25 ~ 0.00)	-0.40 ± 0.31 (-1.25 ~ 0.00)	**.000**
Flat K(D)	42.33 ± 1.18 (40.30 ~ 46.00)	39.39 ± 2.16 (33.60 ~ 43.30)	**.000**
Steep K(D)	44.04 ± 1.13 (42.00 ~ 46.30)	40.30 ± 2.27 (34.60 ~ 43.90)	**.000**
Q value (6 mm)	-0.29 ± 0.22 (-0.58 ~ -0.11)	0.38 ± 0.26 (-0.68 ~ 1.90)	**.000**
tPRK			
Spherical equivalent(D)	-5.21 ± 2.09 (-8.94 ~ -1.12)	-0.12 ± 0.53 (-1.38 ~ 1.25)	**.000**
Spherical diopter(D)	-4.52 ± 2.06 (-8.50 ~ -0.62)	0.09 ± 0.60 (-1.25 ~ 1.75)	**.000**
Cylindrical diopter(D)	-1.37 ± 1.02 (-4.00 ~ 0.00)	-0.43 ± 0.38 (-1.50 ~ 0.00)	**.000**
Flat K(D)	42.77 ± 1.58 (39.20 ~ 45.70)	38.76 ± 2.19 (34.40 ~ 43.00)	**.000**
Steep K(D)	44.70 ± 2.04 (40.60 ~ 49.50)	39.56 ± 2.31 (35.00 ~ 43.80)	**.000**
Q value (6 mm)	-0.37 ± 0.11 (-0.67 ~ -0.17)	0.51 ± 0.48 (-0.45 ~ 1.57)	**.000**
P value§			
Spherical equivalent	0.070	0.060	
Spherical diopter	0.090	0.237	
Cylindrical diopter	0.855	0.657	
Flat K	0.121	0.126	
Steep K	0.138	0.519	
Q value (6 mm)	0.290	**0.023**	

* Paired t test (preoperative vs postoperative)

§ Independent t test (fLASIK vs tPRK)

SimK and 6 mm Q Values were measured by Scheimpflug Tomographer

Data are expressed as mean ± standard deviation and range.

fLASIK, femtosecond laser-assisted in situ keratomileusis; tPRK, transepithelial photorefractive keratectomy; K, keratometry;, SD, standard deviation; D, diopter; SimK, simulated keratometer

Table S2. Results of anterior corneal spherical aberration and total corneal spherical aberration before and after Surgery in Each Area

	Preoperative		Postoperative		*P value**		Change		*P value*§	*P value*¶	
**Diameter**	**Anterior SA**	**Total SA**	**Anterior SA**	**Total SA**	**Anterior SA**	**Total SA**	**Anterior SA**	**Total SA**		**Anterior SA**	**Total SA**
2 mm	0.003718 ± 0.0026150	0.002339 ± 0.004771	-0.00200 ± 0.0064114	0.002259 ± 0.915997	**0.000**	0.813	-0.0057 ± 0.00055	-0.001 ± 0.00825	0.491		
3 mm	0.018847 ± 0.0108358	0.01054 ± 0.0153535	-0.001645 ± 0.0249181	-0.010315 ± 0.0288597	**0.000**	0.093	-0.0205 ± 0.00214	-0.209 ± 0.00274	0.797		
4 mm	0.056891 ± 0.0257765	0.039167 ± 0.0349787	0.027435 ± 0.0552651	-0.003185 ± 0.0931391	**0.000**	**0.023**	-0.0295 ± 0.00495	-0.0424 ± 0.00831	0.050		
5 mm	0.131339 ± 0.0442842	0.096641 ± 0.0524902	0.141159 ± 0.0948798	0.107961 ± 0.1028804	0.232	0.063	0.0098 ± 0.00818	0.0113 ± 0.00964	0.753		
6 mm	0.244766 ± 0.0771245	0.20105 ± 0.083405	0.45343 ± 0.185383	0.396402 ± 0.2359854	**0.000**	0.285	0.2087 ± 0.01540	0.1954 ± 0.02178	0.349		
7 mm	0.395303 ± 0.1274885	0.3509426 ± 0.13110478	1.05431 ± 0.413442	1.062105 ± 0.4451085	**0.000**	**0.000**	0.6590 ± 0.03462	0.7112 ± 0.03803	**0.000**		
				**fLASIK**							
2 mm	0.003587 ± 0.0020393	0.001957 ± 0.0024762	-0.002913 ± 0.0076357	0.016522 ± 0.1484813	**0.022**	0.535	-0.01 ± 0.01	0.01 ± 0.15		0.28	0.30
3 mm	0.018087 ± 0.0085344	0.010500 ± 0.0102432	-0.008065 ± 0.0290933	-0.016348 ± 0.0344624	**0.020**	**0.007**	-0.03 ± 0.03	-0.03 ± 0.03		**0.04**	0.09
4 mm	0.054207 ± 0.0215913	0.036363 ± 0.0247355	-0.001717 ± 0.0576898	-0.024478 ± 0.0668374	0.228	**0.003**	-0.06 ± 0.06	-0.06 ± 0.06		**0.00**	0.09
5 mm	0.129891 ± 0.0389788	0.098978 ± 0.447896	0.062326 ± 0.0785443	0.028174 ± 0.0841381	**0.029**	**0.007**	-0.07 ± 0.08	-0.07 ± 0.08		**0.00**	**0.00**
6 mm	0.241043 ± 0.0854162	0.21152 ± 0.078460	0.29452 ± 0.131622	0.227017 ± 0.1636574	0.102	0.340	0.05 ± 0.14	0.02 ± 0.17		**0.00**	**0.00**
7 mm	0.400209 ±	0.3741061 ± 0.14734814	0.80148 ± 0.362526	0.790630 ± 0.3853122	0.128	0.249	0.40 ± 0.36	0.42 ± 0.39		**0.00**	**0.00**

	0.1457569							
				**tPRK**				
2 mm	0.003795 ± 0.0029115	0.002564 ± 0.0057130	-0.001462 ± 0.0055495	-0.006155 ± 0.0180372	**0.004**	0.381	-0.01 ± 0.01	-0.01 ± 0.02
3 mm	0.019295 ± 0.0120185	0.010564 ± 0.0177549	0.002141 ± 0.0214020	-0.006756 ± 0.0245316	**0.005**	0.604	-0.02 ± 0.02	-0.02 ± 0.03
4 mm	0.058474 ± 0.0279655	0.040821 ± 0.0398672	0.044628 ± 0.0461159	0.009372 ± 0.1039999	**0.021**	0.216	-0.01 ± 0.05	-0.03 ± 0.11
5 mm	0.132192 ± 0.0473575	0.095263 ± 0.0567768	0.187650 ± 0.0698040	0.155015 ± 0.0820024	**0.000**	0.152	0.06 ± 0.07	0.06 ± 0.09
6 mm	0.246962 ± 0.0722816	0.19487 ± 0.086083	0.54714 ± 0.144193	0.496295 ± 0.2146084	**0.000**	0.083	0.30 ± 0.11	0.30 ± 0.21
7 mm	0.392410 ± 0.1162940	0.3372821 ± 0.11941023	1.20341 ± 0.368153	1.222205 ± 0.3996521	**0.000**	**0.000**	0.81 ± 0.31	0.88 ± 0.34

* Paired t test (preoperative vs postoperative)

§ Independent t test (change in Anterior SA vs change in total SA)

¶ Independent t test(fLASIK vs tPRK)

fLASIK, femtosecond laser-assisted in situ keratomileusis; tPRK, transepithelial photorefractive keratectomy; SD, standard deviation
